# Expression of Connexin 43 in Granular Cell Tumors of the Skin, Tongue and Esophagus

**DOI:** 10.3390/dermatopathology10020026

**Published:** 2023-06-16

**Authors:** Hermann Kneitz, Verena Frings, Stefan Kircher, Matthias Goebeler

**Affiliations:** 1 Department of Dermatology, Venereology and Allergology, University Hospital Würzburg, 97080 Würzburg, Germany; 2 Department of Pathology, University Hospital Würzburg, 97080 Würzburg, Germany

**Keywords:** granular cell tumor, connexin 43, immunohistochemistry

## Abstract

Background: Granular cell tumors (GCT) are rare neoplasms of Schwann cell origin occurring in the skin and in other organs. The etiopathogenesis of GCT is yet poorly understood. Connexin 43 (Cx43) is the most broadly expressed gap junction protein in humans, the tumoral role of which has been investigated in several types of tumors. Its role in GCT of the skin, oral cavity and gastrointestinal tract is as yet unknown. Methods: Herein, we present a study on the immunohistochemical expression of Cx43 in GCT of the skin (*n* = 15), tongue (*n* = 4) and esophagus (*n* = 3). Immunolabeling was scored positive (weak (+), moderate (++) or strong (+++)). Results: Cx43 was expressed by all cases of GCT of the skin, tongue and esophagus (22/22), showing moderate to strong staining. All tissue sections of GCT were characterized by a diffuse, cytoplasmic staining pattern of the tumor cells. None of those showed membranous or nuclear staining. Conclusion: Our results suggest that Cx43 probably plays an important role in the development of this rare tumor entity.

## 1. Introduction

Connexins (Cxs) are transmembrane proteins that form gap junctions, which are specialized structures that allow direct intercellular communication between adjacent cells [1]. Cxs play a key role in maintaining cellular homeostatic balance and are involved in a variety of cellular processes, including cell proliferation, differentiation, and cell death [2]. In recent years, several Cxs have been studied in different tumor types, focusing on their pro- and antitumor properties. In carcinogenesis, Cxs have long been considered as tumor suppressors [3], but they may also be involved in tumor invasion. Therefore, Cxs play a context-dependent role in tumors, and their expression pattern may change during tumor development and progression [4]. Cx43, a 43 kDa protein, is the most abundant member of the Cx family in humans; it is expressed in both the epidermis and dermis [1]. At present, there is evidence that Cx43 may play an important role in the development of skin tumors [5]. Thus, elevated levels of Cx43 have been demonstrated in various types of primary skin tumors (squamous cell carcinoma, basal cell carcinoma, trichoblastoma, and melanoma [6,7,8]. Control or reference tissues in these studies included regular epidermis, regular skin adnexa, and nevi. Additionally, elevated levels of Cx43 have been observed in neoplasms of other organ systems, such as breast, colon and laryngeal carcinoma [9,10,11]. We recently were able to demonstrate an increased, conspicuous aberrant expression of Cx43 in Kaposi’s sarcoma and angiosarcomas, while benign hemangiomas showed no Cx43 expression [12]. Cx43 is probably the best-studied Cx in tumorgenesis [1]; however, we have not found any previous studies of Cx43 expression in granular cell tumors (GCT). GCT is a rare benign neoplasm that was originally described by Abrikossoff in 1926 [13]. Due to their nonspecific presentation, these tumors are often misdiagnosed clinically. Histopathological analysis is mandatory for correct diagnosis. GCT are usually benign tumors with Schwann cell differentiation. Malignant progression is seen in approximately 2% of cases [14]. GCT accounts for 0.5% of all soft-tissue tumors [15]. Female preponderance is described and GCT have been reported to mostly occur between the third and fifth decades of life [16]. In 30–45% of cases, GCT affects the skin. The second most frequent localization is the oral cavity with the tongue [17]. Less frequently, GCT arises in the gastrointestinal tract, where the esophagus is the most common site. Histomorphologically, GCTs are usually indistinctly circumscribed and lack a capsule. Tumor cells are relatively large and irregularly shaped, cytoplasm is granular and atypia is absent. The reason for the granular changes is unclear; a reactive or degenerative response has been suggested. Electron microscopy revealed that the granules represent remnants from degenerated myelinated axons [18].

The purpose of this study was to analyze the expression and localization of Cx43 as a marker of gap junctions in 22 GCTs of the skin, tongue and esophagus.

## 2. Materials and Methods

For our retrospective study, 22 cases of GCT were retrieved from the archives of the Department of Dermatology, University Hospital Würzburg, and the Institute of Pathology, University of Würzburg, and their clinical data were collected from the patients’ files. The study was designed following the Declaration of Helsinki of Ethical Principles for Medical Research involving Human Subjects and approved by the Ethics Committee of the University of Würzburg.

Sections of formalin-fixed, paraffin-embedded tissue, 4 μm thick, were deparaffinized and rehydrated, pretreated with an epitope retrieval solution (Target Retrieval Solution, pH 9 (S2367, Dako, Hamburg, Germany); diluted 1:10 in distilled aqua) and then incubated with an affinity-purified rabbit polyclonal serum against human Cx43 (#C6219, Sigma-Aldrich, Darmstadt, Germany; dilution 1/4000) using the Dako Autostainer Plus. Staining was performed applying the Dako REAL Detection System Alkaline Phosphatase/RED (#K5003 Dako, Hamburg, Germany). All paraffin-embedded sections were blinded and independently evaluated by two dermatohistopathologists (HK and VF). When evaluating the immunohistochemistry, we distinguished between weak (+), moderate (++) or strong (+++) staining intensity. We also made a distinction between diffuse staining (when the entire tumor was stained) and focal staining (when some tumoral areas were not stained). Immunohistochemical evaluation was performed on five representative sections of each tumor at 200× magnification. Nodular basal cell carcinomas and normal epidermis served as positive controls, and placenta and tonsillar tissue as negative controls, for anti-Cx43 staining. Additional immunohistochemistry was performed using antibodies against the following targets: S100B (polyclonal, Dako, Groschup, Denmark), SOX10 (clone EP 268, Cell Marque, Rocklin, CA, USA), NSE (clone BBS/NC/VI-H14, Dako, Groschup, Denmark), actin (clone1A4, Dako, Groschup, Denmark), desmin (clone D33, Dako, Groschup, Denmark), and pan-cytokeratin (AE1/AE3, Dako, Groschup, Denmark).

## 3. Results

A total of 22 specimens of GCT were included in the study, and their clinical data are summarized in Table 1. The gender distribution of cutaneous GCT was balanced, with an equal number of male and female patients. The average age at the time of primary diagnosis was 41.1 years, ranging from 4 to 71 years. Among the cases, eight GCTs were located on the trunk, five on the extremities, and one each on the lip and perianal area. All patients with GCT of the tongue and esophagus were females. 

**Figure 1:**
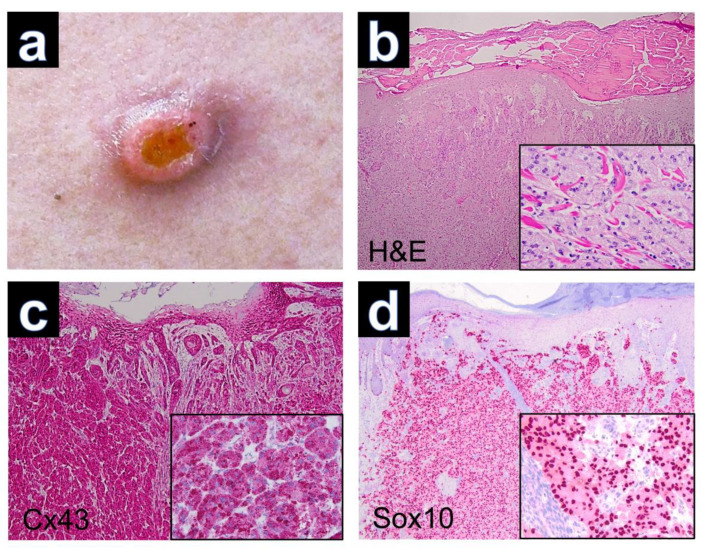
(**a**) Granular cell tumor with exophytic growth and central erosion. (**b**) Reactive hyperplasia of the epidermis (H&E, ×40) and tumor cells with trabecular growth pattern (inset, H&E, ×200). (**c**) Expression of Cx43 in regular keratinocytes of the epidermis and positive staining of Cx43 in dermal parts of the GCT (Cx43, ×40). Note the cytoplasmic immunostaining of tumor cells (inset, ×200). (**d**) Expression of Sox10 with nuclear staining of tumor cells (Sox10, ×40), (inset, ×200).

Tumor excision was performed in all patients. Clinical imaging was only available for case GCT-12, which presented as a solitary tumor nodule with superficial erosion (Figure 1a). The hematoxylin-eosin-stained sections of GCT demonstrated nests and sheets of large polygonal cells with abundant eosinophilic, granular cytoplasm and poorly defined cell borders in all cases. The nuclei were round and hyperchromatic with inconspicuous nucleoli (Figure 1b). Reactive pseudoepitheliomatous hyperplasia was seen in several cases of cutaneous GCTs, which had to be differentiated from primary squamous cell carcinoma of the skin. Upon PAS staining, the tumor cells showed PAS-positive intracytoplasmic granules, which are thought to represent atypical phagolysosomes [17]. 

**Figure 2:**
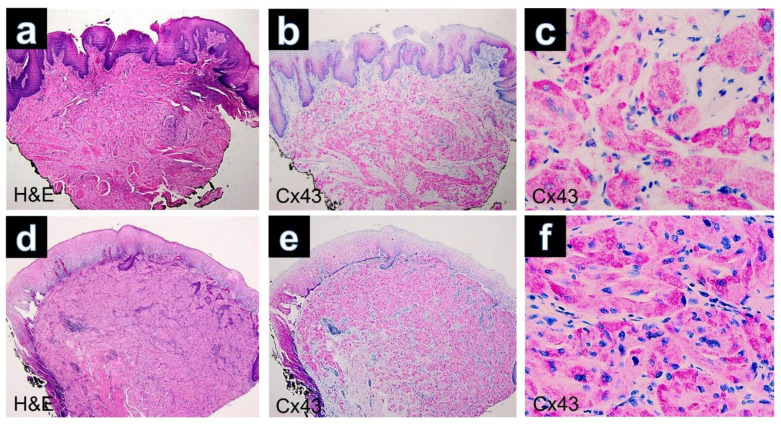
(**a**) GCT of the tongue. Poorly defined lesion composed of sheets separated by collagenous bands (H&E, ×40). (**b**) Immunohistochemistry for Cx43 showing diffuse positivity in the lesional cells (×40). (**c**) Cytoplasmatic positivity of the tumors cell by immunostaining with Cx43 (Cx43, ×400). (**d**) GCT of the esophagus. The tumor is well circumscribed and infiltrates the muscularis mucosae (H&E, ×40). (**e**) Expression of Cx43 in regular keratinocytes of the superficial epithelium and positivity of Cx43 in dermal parts of the GCT (Cx43, ×40). (**f**) Cytoplasmic positivity of the tumor cells observed in the immunostaining for Cx43 (×400).

**Figure 3:**
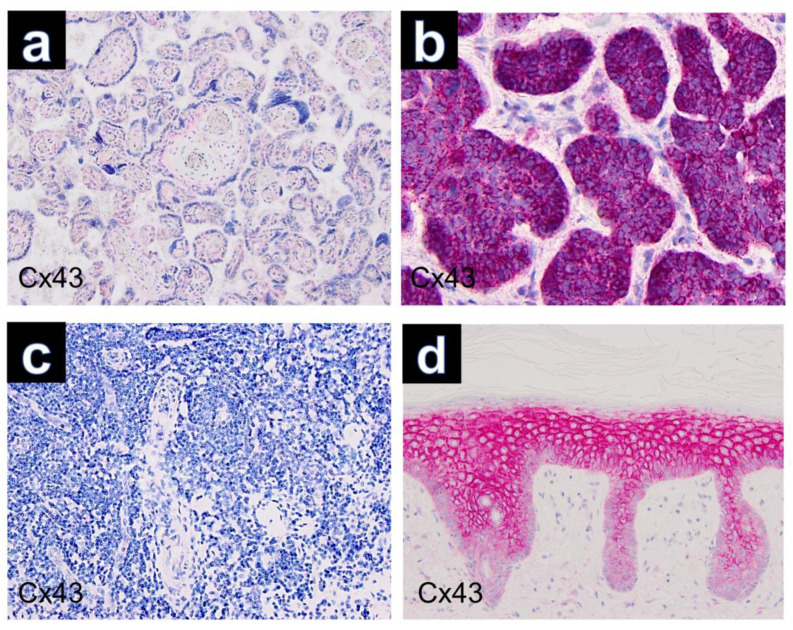
Controls used in this study (Cx43, ×100): (**a**) placenta, (**b**) basal cell carcinoma, (**c**) tonsillar tissue, (**d**) healthy skin.

Immunohistochemically, all cutaneous GCTs (15/15) showed cytoplasmic positivity for Cx43, predominantly with strong staining intensity (Figure 1c). Additionally, positive staining was observed for Sox 10 (Figure 1d), S100, CD68 and neuron-specific enolase (NSE) in all GCTs. GCTs of the tongue and esophagus also exhibited marked cytoplasmic positivity for Cx43 by immunolabeling (Figure 2). The results of immunohistochemical staining for Cx43 in all examined GCTs are summarized in Table 1. Among the cutaneous GCT cases, the majority (10/15) (66.6%) demonstrated strong cytoplasmic staining (+++), while in 5 out of 15 cases (33.3%), the staining intensity was moderate (++). Normal placenta, normal tonsillar tissue, solid basal cell carcinoma and healthy skin were used as controls (Figure 3). Solid basal cell carcinoma and normal epidermis both showed high expression of Cx43, characterized by a membranous pattern. In the epidermis of normal skin, Cx43 staining was minimal in basal keratinocytes and more prominent between the keratinocytes of the spinous and granular layers (Figure 3d). Additionally, immunostaining for Cx43 was also detected in hair follicles, sebaceous glands, and eccrine sweat ducts. 

Other tumors (neurofibroma, leiomyoma, dermatofibroma, xanthoma, xanthogranuloma and Spitz nevus) that are important in the differential diagnosis of granular cell tumors were also examined. Immunohistochemical analysis revealed negative results for immunostaining of Cx43 in these tumors. 

## 4. Discussion

GCTs were first described in 1926 by the Russian pathologist Alexei Iwanowitsch Abrikossoff (1875–1955) [13]. They were initially coined granular cell myoblastomas, as they were believed to be of muscular origin. With the advent of immunohistochemical stains and electron microscopy, they are now considered to be of Schwannian derivation. The etiopathogenesis of GTCs, however, is yet poorly understood. 

In our cohort of cutaneous GCT, the mean age at primary diagnosis corresponded with the findings reported in the existing literature. However, unlike some other studies, we did not observe a gender dominance in our cohort [19,20]. The localization of cutaneous GCT in the trunk and extremities was similar to the data published in previous studies [21,22,23]. 

Histologically, well-demarcated proliferations of pale-stained, polygonal to spindle-shaped cells with characteristic eosinophilic granular cytoplasm and oval nuclei were observed in all GCT samples of our cohort. The diagnosis of GCT was confirmed by immunohistochemical staining for S100, Sox10, NSE and CD68. S100 is the most commonly used diagnostic marker demonstrating the Schwannian origin of GCT [24]. However, single cases of S100-negative GCTs have also been described [25]. CD68-positivity of tumor cells is attributed to intracytoplasmic accumulation of phagolysosomes and does not reflect a histiocytic genesis of the tumor [26]. In a variety of benign and malignant tumors, focal or more extensive granular changes of the cytoplasm may sometimes also exist. In these cases, granular cell differentiation is mostly caused by an increased number of secondary lysosomes [27]. 

Occasionally, neurofibromas and schwannomas may exhibit granular changes in limited parts of the tumor. However, these changes are never present throughout the entire tumor, so they do not pose a diagnostic challenge. In contrast, basal cell carcinoma, melanoma, xanthoma, leiomyoma, leiomyosarcoma, dermatofibrosarcoma, angiosarcoma, dermatofibroma and ameloblastoma can have granular cell variants in a pathological context, which can be distinguished from GCT through immunohistochemical studies.

Cxs are typically found on the cell surface as membrane proteins that form intercellular gap junctions (GJs), enabling rapid communication between adjacent cells through the direct exchange of small molecules, electrical signals, and ions [28]. Previous studies have suggested that atypical cytoplasmic localization of Cxs and the consequent disruption of gap junction-mediated intercellular communication (GJIC) may play a significant role in tumor progression, invasion, and metastasis in various malignancies, including carcinomas, melanoma and leukemia [29,30,31,32]. The components of Cxs are stored in the cytoplasm, and impaired trafficking reduces the uptake of Cxs into the cell membrane from the cytoplasm [28,32,33].

Connexin 43 (Cx43) is a protein that forms GJs in various tissues, including the skin. In the skin, Cx43 has been implicated in various processes, including wound healing, epidermal differentiation and melanogenesis. There is evidence to suggest that alterations in Cx43 expression and function may play a role in the development and progression of skin tumors. Furthermore, experimental studies have demonstrated that restoring Cx43 expression in skin cancer cells can inhibit tumor growth and promote apoptosis [34]. 

Cx43 normally forms channels in the cell membrane, leading to a membranous staining pattern when labeled with an anti-Cx43 antibody. However, in GCT, the staining is only detected in the cytoplasm of tumor cells. This suggests that the abnormal cytoplasmic expression of Cx43 in GCT may be associated with a defect in the GJ assembly, resulting in increased retention of Cx43. The retention of Cx43 and the subsequent lack of GJIC have previously been observed in gastric, pancreatic, breast and lung cancers [35,36,37,38,39] and also in skin tumors such as atypical fibroxanthoma [40], and more recently in Kaposi’s sarcoma and angiosarcoma [12]. This observation may point to an important functional role of Cx43 in carcinogenesis, and some authors have therefore suggested that aberrant Cx43 expression could promote oncogenesis [8,41]. In addition, aberrant cytoplasmic expression observed upon routine immunohistochemical staining is not uncommon in the case of other oncoproteins. For example, C-kit is expected to show membranous staining under normal circumstances; however, a cytoplasmic and globular/dot-like staining is also frequently encountered in certain tumor entities [42].

Another possible explanation for the aberrant cytoplasmic localization of Cx43 in GCT could be its accumulation in phagolysosomes. Several studies reported a physiological accumulation of Cx within lysosomes, so that in GCT a disturbed degradation of Cx43 within phagolysosomes seems conceivable [43,44,45]. 

This hypothesis is further supported by recent findings of ATP6AP1 or ATP6AP2 gene mutations in cutaneous GCT [46]. ATP6AP1 and ATP6AP2 encode V-ATPases, which regulate cellular pH, especially in lysosomes [47]. Mutations in these V-ATPase genes can lead to a decrease in cellular acidity, resulting in impaired lysosomal degradation and the non-physiological accumulation of Cx43 in phagolysosomes. The cytoplasmic accumulation of Cx43 in GCT may thus provide an explanation for the observed immunohistochemical staining pattern. Further research is needed to elucidate the precise mechanisms underlying the aberrant cytoplasmic expression of Cx43 in GCT and its implications in tumor development and progression. 

## 5. Conclusions

The marked expression of Cx43 in GCT, which was detectable in all cases of our cohort, suggests that Cx43 probably plays an important role in the development of these tumors. Our observations may contribute to our understanding of GCT. Additional studies are needed both to understand the molecular pathological role of Cx43 in the development of GCT and to extend the studies to malignant and nonneuronal GCT, which were not included in the present study.

## Figures and Tables

**Table 1:** Patient characteristics and immunohistochemical expression of Cx43.

Entity	Age [Years]	Sex	Localization	Pattern	Intensity
GCT-1	4	M	Perianal	D, C	strong
GCT-2	39	F	Lip	D, C	strong
GCT-3	23	M	Forearm	D, C	strong
GCT-4	18	F	Trunk	D, C	moderate
GCT-5	71	F	Thigh	D, C	moderate
GCT-6	49	F	Thigh	D, C	strong
GCT-7	26	F	Breast	D, C	moderate
GCT-8	8	F	Hand	D, C	strong
GCT-9	58	M	Neck	D, C	strong
GCT-10	64	M	Neck	D, C	moderate
GCT-11	40	M	Back	D, C	strong
GCT-12	44	M	Back	D, C	strong
GCT-13	50	F	Breast	D, C	moderate
GCT-14	52	F	Trunk	D, C	strong
GCT-15	71	M	Leg	D, C	strong
GCT-16	40	F	Tongue	D, C	moderate
GCT-17	41	F	Tongue	D, C	strong
GCT-18	42	F	Tongue	D, C	moderate
GCT-19	23	F	Tongue	D, C	moderate
GCT-20	48	F	Esophagus	D, C	moderate
GCT-21	51	F	Esophagus	D, C	strong
GCT-22	23	F	Esophagus	D, C	moderate

Abbreviations: GCT, granular cell tumor; F, female; M, male; D, diffuse; F, focal; C, cytoplasmatic; M, membranous. Note: Age at time of the biopsy [years].

## Data Availability

Not applicable.
